# Anticancer effect of the antirheumatic drug leflunomide on oral squamous cell carcinoma by the inhibition of tumor angiogenesis

**DOI:** 10.1007/s12672-025-01763-5

**Published:** 2025-01-16

**Authors:** Chieko Niwata, Takayuki Nakagawa, Takako Naruse, Miyuki Sakuma, Nao Yamakado, Misaki Akagi, Shigehiro Ono, Kei Tobiume, Jing Gao, Eijiro Jimi, Kouji Ohta, Tomonao Aikawa

**Affiliations:** 1 Department of Oral and Maxillofacial Surgery, Graduate School of Biomedical and Health Sciences, Hiroshima University, 1-2-3, Kasumi, Minami-ku, Hiroshima, 734-8553 Japan; 2 Graduate School of Biomedical & Health Sciences (Dentistry & Oral Health Sciences), Hiroshima University, 1-2-3, Kasumi, Minami-ku, Hiroshima, 734-8553 Japan; 3 Laboratory of Molecular and Cellular Biochemistry, Faculty of Dental Science, Kyushu University, 3-1-1 Maidashi, Higashi-ku, Fukuoka, 812-8582 Japan; 4 Department of Public Oral Health, Graduate School of Biomedical and Health Sciences, Hiroshima University, 1-2-3, Kasumi, Minami-ku, Hiroshima, 734-8553 Japan

**Keywords:** Leflunomide, Oral squamous cell carcinoma, Syngeneic mice, Drug repositioning

## Abstract

**Objectives:**

Leflunomide (LEF) is a conventional synthetic disease-modifying antirheumatic drug and suppresses T-cell proliferation and activity by inhibiting pyrimidine synthesis using dihydroorotase dehydrogenase (DHODH); however, several studies have demonstrated that LEF possesses anticancer and antiangiogenic effects in some malignant tumors. Therefore, we investigated the anticancer and antiangiogenic effects of LEF on oral squamous cell carcinoma (OSCC).

**Methods:**

To evaluate the inhibitory effect of LEF on OSCC, cell proliferation and wound-healing assays using human OSCC cell lines were performed. The DHODH inhibitory effect of LEF was evaluated by Western blot. To assess the suppression of pyrimidine biosynthesis induced by LEF on OSCC, cell proliferation assays with or without uridine supplementation were performed. The antiangiogenic effect of LEF was evaluated by in vitro tube formation assay using immortalized human umbilical vein endothelial cells, which were electroporatically transfected with hTERT. The tumor-suppressive effect of LEF in vivo was examined in both immunodeficient and syngeneic mice by implanting mouse OSCC cells. Tumor vascularization was evaluated by immunohistochemistry of the tumor extracted from syngeneic mice.

**Results:**

LEF dose-dependently inhibited OSCC proliferation and migration. LEF significantly inhibited DHODH expression, and uridine supplementation rescued the inhibitory effect of LEF. LEF dose-dependently suppressed endothelial tube formation. In the animal study, LEF significantly suppressed tumor growth in both immunodeficient and syngeneic mice. Histologically, LEF decreased DHODH expression and tumor vascularization.

**Conclusion:**

LEF is a potent anticancer agent with antiangiogenic effects on OSCC and might be clinically applicable to OSCC by drug repositioning.

**Supplementary Information:**

The online version contains supplementary material available at 10.1007/s12672-025-01763-5.

## Introduction

Approximately 370,000 new cases of oral cancer and 170,000 related deaths occur annually worldwide [1]. The 5-year survival rate for oral cancer ranges from 50 to 60% [2]. Surgery is the first-line treatment for oral cancer; however, some patients with comorbidities should avoid surgery. These patients require radiotherapy and chemotherapy [3, 4]. Traditional cytotoxic anticancer agents such as platinum and taxane agents have been proven effective against oral cancer and are widely used clinically [3–8]. In addition, anti-epithelial growth factor receptor (EGFR) antibodies such as cetuximab [9] and immune checkpoint inhibitors such as nivolumab [9, 10] and pembrolizumab [12–15] have been introduced as novel agents against oral cancer, which contribute to improved survival rates. However, there are fewer anti-cancer drugs available for oral cancer compared to other cancers, and the currently indicated drugs have limited mechanisms of action. There is an urgent need to develop drugs with novel mechanisms of action.

Leflunomide (LEF) is an immunosuppressive agent that inhibits pyrimidine synthesis using dihydroorotase dehydrogenase (DHODH) and is widely used in the treatment of rheumatoid arthritis [16, 17]. The demand for pyrimidines, components of nucleic acids, is increased in activated T-lymphocytes [18]. Pyrimidine biosynthesis is mediated by de novo and salvage pathways; however, activated T-lymphocytes are more dependent on the de novo pathway [19, 20]. LEF reversibly inhibits the activity of DHODH, a key enzyme in the de novo synthesis of pyrimidine nucleotides, causing the arrest of activated lymphocytes in the G1 phase of the cell cycle [18, 21]. Consequently, T-lymphocyte function is suppressed, exerting an immunosuppressive effect.

On the other hand, the inhibitory effect of LEF on pyrimidine synthesis induces a favorable anti-tumor effect. Inhibition of pyrimidine supply has a growth-inhibiting effect in malignant tumors. Therefore, LEF has been examined in preclinical studies in various malignant tumors [22–27]. However, inhibition of pyrimidine synthesis is not the only pharmacological action cited as an anti-tumor effect of LEF. Several studies have demonstrated that LEF inhibited the activity of tyrosine kinases such as EGFR and platelet-derived growth factor receptor [28–30]. Other studies have focused on the inhibitory effect on tumor angiogenesis [31, 32]. These studies suggest that LEF has the potential to be repositioned as an effective anti-cancer agent.

In the present study, we aimed to reposition LEF as an antirheumatic agent to a novel anticancer agent for oral cancer. Therefore, the anticancer effects of LEF on oral squamous cell carcinoma (OSCC) and tumor angiogenesis inhibition in vitro were investigated. LEF is essentially classified as an immunosuppressant, and if only its inhibitory effect on pyrimidine metabolism is considered, it may exert a negative effect on T-cell-dependent tumor immunity. Therefore, using two mouse models of immunodeficient and syngeneic, this study investigated whether the anticancer effect of LEF is affected by the presence or absence of T cells.

## Materials and methods

### Cell culture and reagents

Human OSCC (hOSCC) cell lines SAS (JCRB0260), Ca9-22 (JCRB0625), HSC2 (JCRB0622), and HSC3 (JCRB0623) were purchased from the Japanese Collection of Research Bioresources (JCRB) Cell Bank (Osaka, Japan). All hOSCC cells were cultured in 5% CO_2_ at 37 °C and maintained in Dulbecco’s modified eagle medium (Sigma-Aldrich, St. Louis, MO, USA) with 10% fetal bovine serum (FBS) and 100 IU/mL penicillin/100 µg/mL streptomycin (Nacalai Tesque, Kyoto, Japan).

The mouse OSCC (mOSCC) cell line SCC VII was provided by Prof. Jimi of Kyushu University. The cell culture environment was the same as that for the hOSCC lines, as described above. The cells were maintained with RPMI1640 culture medium (Sigma-Aldrich, St. Louis, MO, USA) with 10% FBS and 100 IU/mL penicillin/100 µg/mL streptomycin (Nacalai Tesque, Kyoto, Japan).

HUEhT-1 (JCRB1458) cells were also purchased from JCRB Cell Bank. The cells were cultured in 5% CO_2_ at 37 °C and maintained with an endothelial cell growth medium (Promo Cell, Heidelberg, Germany) with 2% FBS, 0.5 ng/mL recombinant human vascular growth factor-165, 10 ng/mL recombinant human basic fibroblast growth, 5 ng/mL recombinant human epidermal growth factor, 20 ng/mL insulin-like growth factor, 1.0 µg/mL ascorbic acid, 22.5 µg/mL heparin, and 0.2 µg/mL hydrocortisone (Promo Cell Promo Cell, Heidelberg, Germany). Before culture, plates and dishes were covered with 0.5 µg/cm^2^ vitronectin (A14700, Gibco, Carlsbad, CA, USA). LEF was purchased from Tokyo Chemical Industry Co., Ltd. (Tokyo, Japan). A stock solution of LEF was reconstituted with dimethyl sulfoxide (DMSO, Sigma-Aldrich, St. Louis, MO, USA). The stock solution was diluted with a culture medium before use and the final concentration of DMSO was 0.1%.

### Cell proliferation assay

Cell viability was evaluated by water-soluble formazan using Cell Counting Kit-8 (Dojindo Laboratories, Kumamoto, Japan) according to the manufacturer’s instructions. Each cell line was seeded in 96-well plates at 3.0 × 10^3^ cells /well. After the cells were incubated for 24, 48, or 72 h with 0 (control), 1.0 µM, 10 µM, 100 µM at the respective LEF concentrations, the kit reagent WST-8 was added to the medium and incubated for another 2 h. Sample absorbance (450 nm) was determined using the 800TS™ Absorbance Microplate Reader (BioTek Instruments Inc., Winooski, VT, USA). Twelve-well replicates were analyzed for each incubation time and LEF concentration condition.

### Colony formation assay

Cells from each OSCC line were seeded in 6-well plates at 2.5 × 10^3^ cells /well. After 10 days of incubation, the cells were fixed with 70% ethanol and stained with crystal violet for 30 min. The number of colonies (> 2 mm) on the images was counted using Image J (US National Institutes of Health, Bethesda, MD, USA).

### Wound-healing assay

Cells from each OSCC line were seeded in 6-well plates at 1.0 × 10^6^ cells /well and cultured until achieving a confluent monolayer. The cells were scratched with the tip of a 200-µL pipette. After washing with phosphate-buffered saline (PBS), the cells were incubated under the indicated concentration of LEF for 12 h. To determine the size of the scratches before and after incubation, the same area was observed under a phase-contrast microscope (BZ-X810; KEYENCE Corporation, Osaka, Japan), and the reduction rate in the scratched area was calculated from the equation R = (A_1_ – A_2)_ /A_1,_ where A1 and A2 indicate the area immediately after scratching and the area after 12 h, respectively.

### Lactate dehydrogenase (LDH) assay

The LDH assay was performed to investigate LEF cytotoxicity using the Cytotoxicity LDH Assay Kit (Dojindo Laboratories, Kumamoto, Japan). Cells from each OSCC line were incubated in 96-well plates under the indicated LEF concentration for 24 h. Then, the harvested supernatant of the culture medium was mixed with reagents following the manufacturer’s protocol. Sample absorbance (490 nm) was measured using 800TS™ Absorbance Microplate Reader (BioTek Instruments Inc., Winooski, VT, USA). The amount of released LDH was assessed by calculating the ratio of the absorbance at each concentration of LEF to the absorbance of control.

### Western blotting

Cells from each hOSCC line were seeded in 10-cm dich at 5.0 × 10^6^ cells / dish and cultured for 48 h under the indicated LEF concentration. Harvested cells were washed with PBS. Protein was extracted from each sample using a RIPA Buffer (Nacalai Tesque, Kyoto, Japan). Quantification of the extracted proteins was performed by the BCA method using Pierce™ BCA Protein Assay Kits (Thermo Fisher Scientific Inc, Waltham, Massachusetts, USA). Western blotting was performed as described previously [33]. Briefly, the protein was electrophoresed in 10% sodium dodecyl sulfate–polyacrylamide gel and transferred onto a polyvinylidene difluoride membrane. Primary antibodies, namely, DHODH rabbit pAb (14877-1-AP; at 1: 1000, Proteintech, Rosemont, IL, USA) and GAPDH mouse mAb (#MAB374 at 1:2000; Millipore, Billerica, MA, USA), were used. DHODH expression was visualized on the LAS 4000 Mini-Imaging System (Fujifilm, Tokyo, Japan) and quantified by calculating the DHODH/GAPDH ratio using Image J (US National Institutes of Health).

### Uridine supplementation assay

Each cell line was seeded in 96-well plates at 3.0 × 10^3^ cells /well. Twelve-well replicates were prepared for analysis. The cells were incubated for 72 h, with 0 (control), 1.0 µM, 10 µM, 100 µM at the respective LEF concentrations with or without 100 µM uridine (Tokyo Chemical Industry Co., Ltd., Tokyo, Japan). The kit reagent WST-8 was added to the medium and incubated for another 2 h. Sample absorbance (450 nm) was determined using the 800TS™ Absorbance Microplate Reader (BioTek Instruments Inc., Winooski, VT, USA).

### Tube formation assay

The tube formation assay was performed as described previously [33]. The assay was performed using In Vitro Angiogenesis Assay Kit Tube Formation (R&D Systems, Minneapolis, MN, USA) according to the manufacturer’s protocol. Briefly, HUEhT-1 cells were pretreated with calcein, suspended in the serum-free culture medium containing the indicated concentration of LEF and 100 ng/mL VEGF_165_ (Peprotech, Rocky Hill, NJ, USA), and then seeded in 96-well glass-bottom plates coated by vitronectin at a cell concentration of 2.0 × 10^4^ cells/well. As an inhibitor control, sulforaphane was added to the reference cell at a final concentration of 10 µM. After 12 h, a fluorescent staining dye was applied to each well. The cells were observed with BZ-X810 in the bright-field and fluorescent (FTIC/eGFP) field. The number of junctions, number of meshes, number of segments, and total length of the segments were evaluated on the images using Image J (Angiogenesis Analyzer for Image J, US National Institutes of Health).

### Animal experiments

The animal experimentation protocol was subjected to review and approval by the Review Board of the Animal Experimentation Committee of Hiroshima University (Approval No. A21-135). Ten each of 4-week-old female BALB/c-nu/nu and 5-week-old female C3H/HeN Jcl mice (CLEA Japan, Inc., Tokyo, Japan) were housed in a temperature- and humidity-controlled facility under a 12-h light–dark cycle. Mice had ad libitum access to food and water. BALB/c and C3H mice were divided into LEF-treated and control groups of 5 mice each, and were inoculated subcutaneously with 5.0 × 10^5^ cells of SCC VII for both allograft and syngeneic mOSCC models. The LEF was dissolved in 2% carboxymethyl cellulose and administered orally and started at the same time as the tumor inoculation. The LEF dose was 20 mg/kg/day every day for 14 days. Control group received 0.1% DMSO which dissolved in 2% carboxymethyl cellulose orally for 14 days. Fourteen days after tumor inoculation, mice were sacrificed, and tumors were harvested. The tumor weight was measured with a digital balance (Sartorius Entris 5201-1S; Göttingen, Germany), and the tumor volume was calculated from the equation V = R_1_ / 2 × R_2_^2^, where R1 and R2 indicate the longitudinal radius and radius measured with a caliper, respectively.

### Histological examinations

Syngeneic tumor specimens were fixed in Periodate-Lysine-Paraformaldehyde (PLP) fixative, and embedded in paraffin, and sectioned at 4.5 μm thickness using a microtome. Sections were deparaffinized with xylene, and dehydrated with ethanol with concentration gradient (100% ethanol 3 min, 100% ethanol 3 min, 95% ethanol 3 min). Sections stained with hematoxylin and eosin (HE) in accordance with standard protocols. For Immunohistochemistry (IHC), sections were incubated with 3% H_2_O_2_ solution for 45 min at room temperature, and treated with Tris/EDTA for antigen retrieval. Sections were incubated with 3% BSA at 37 °C for 30 min for blocking. Immunostaining was performed using primary antibodies against vascular endothelial growth factor receptor 2 (VEGFR2) (rabbit mAb, 55B11; at 1: 100, Cell Signaling Technology, Danvers, MA, US), integrin alpha V rabbit mAb (ab179475; at 1: 500), CD31 mouse mAb (ab182981; at 1: 2000, Abcam Inc., Cambridge, MA, USA), DHODH rabbit pAb (14877-1-AP; at 1: 300, Proteintech, Rosemont, IL, USA). After incubation with primary antibodies at 4 °C for 12 h, sections were incubated with the secondary antibodies with Signal Stain Boost IHC Detection Reagent (HRP, Rabbit) (Cell Signaling Technology, Danvers, MA, USA) at room temperature for 1 h. After DAB and hematoxylin staining, the sections were sealed with neutral resin and visualized by phase-contrast microscopy (BZ-X810; KEYENCE Corporation, Osaka, Japan). Quantitative analysis of stained area of each section was performed by using BZ-X Analyzer software (BZ-H4A, KEYENCE Corporation, Osaka, Japan). Positive rate was defined as the ratio of the staining area of each marker to the horizontal projected area of the section; five sections were randomly selected from each marker and their respective staining rates were calculated.

### Statistical analysis

All experiments were repeated at least three times. Statistical analysis was performed using Student’s t-test with JASP version 0.18.3 (JASP Team (2024)). Results were expressed as means ± standard errors of mean (SEM). Differences at *p* < 0.05 were considered significant.

## Results

### Inhibitory effect of LEF on human OSCC proliferation and migration

**Fig. 1:**
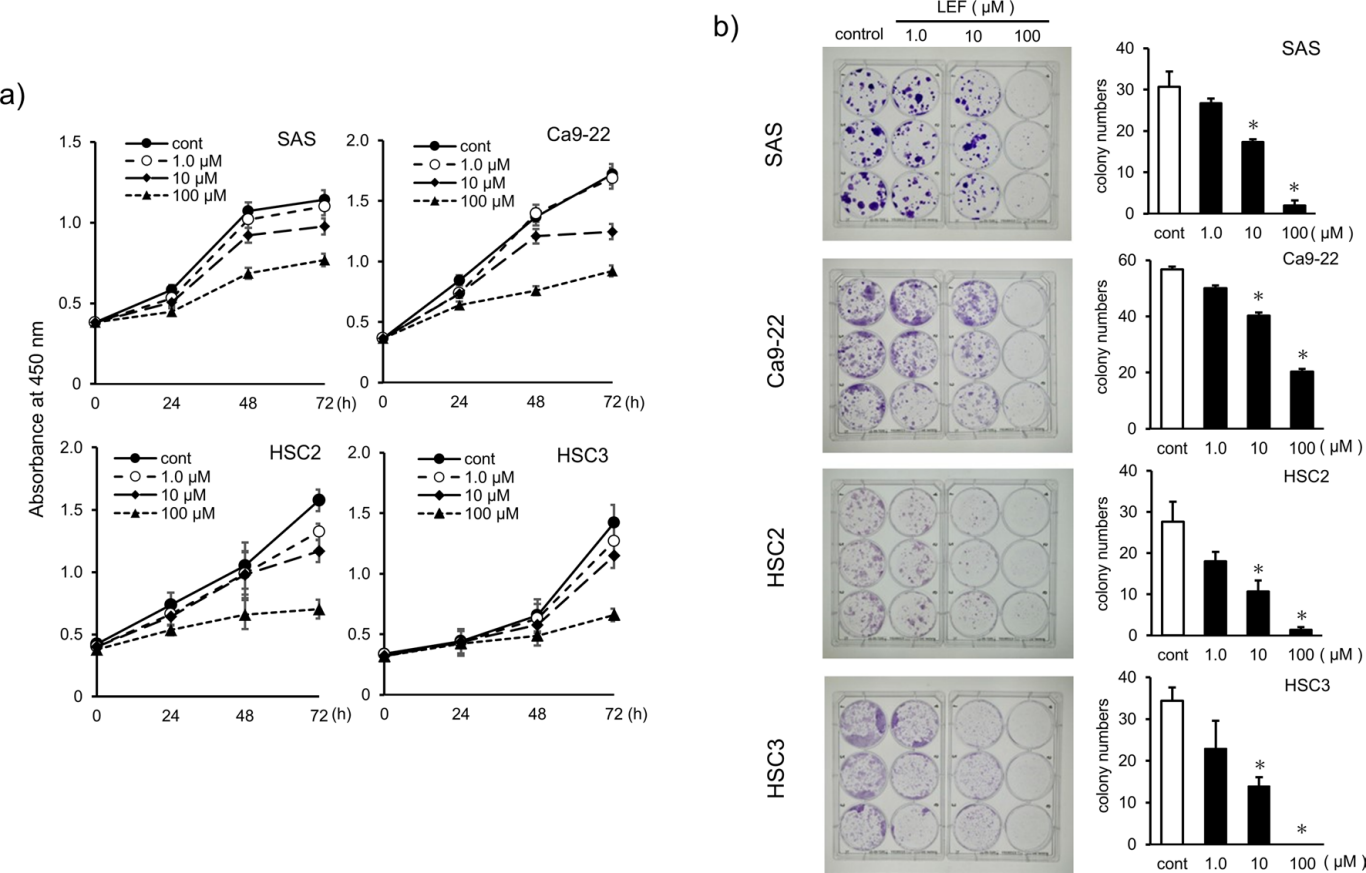
Effects of leflunomide (LEF) on the proliferation and migration of human OSCC cell lines. **a** Effect of LEF on the growth of human OSCC cell lines SAS, Ca9-22, HSC2, and HSC3. OSCC cells were treated with the indicated amount of LEF or dimethyl sulfoxide (DMSO) as a control for 24, 48, and 72 h, and the CCK-8 assay was performed. **b** The colony formation assay was also performed to determine the effect of LEF on proliferation into OSCC cells. The left panel shows representative pictures in each cell line, whereas the right panel shows a quantitative analysis of colony formation. Each experiment was performed > 5 times and obtained similar results. Statistical analysis was performed using Student’s t-test. Values are presented as means ± SEM versus control (*p < 0.05)

**Fig. 2:**
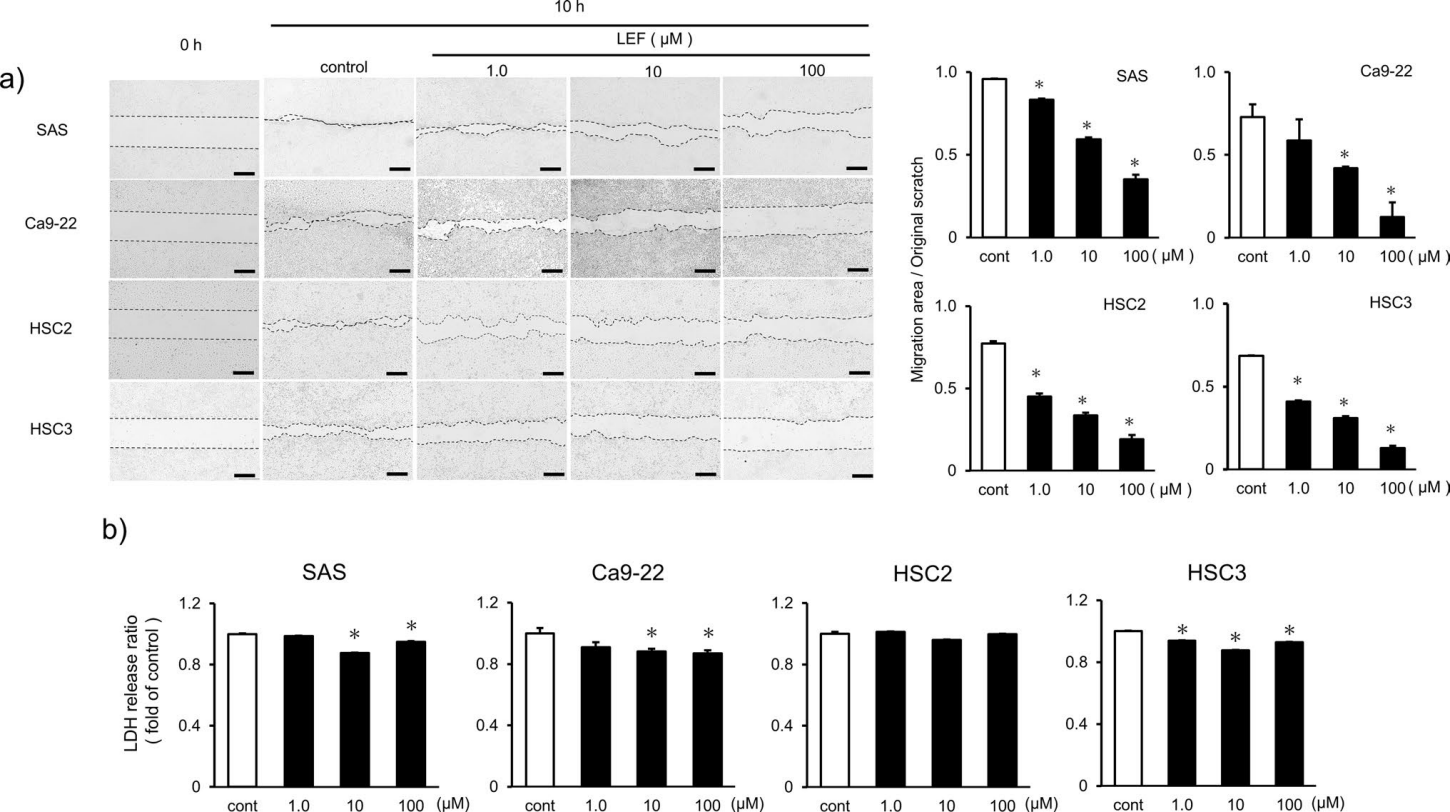
Effects of LEF on the migration and cytotoxicity against human OSCC cell lines. **a** Wound-healing assay of LEF-treated OSCC cells. The panels show representative figures at each concentration of LEF. Black bars indicate 200 µm. The graph shows the reduction rate for each LEF concentration relative to the original scratch area. **b** LEF cytotoxicity against OSCC cells. OSCC cells were incubated with the indicated concentration of LEF or DMSO for 24 h, and the amount of lactate dehydrogenase (LDH) in the culture medium was measured according to the manufacturer’s protocol of the Cytotoxicity LDH Assay Kit (Dojindo). Sample absorbance (490 nm) was measured by microplate reader. To evaluate released LDH, the value of absorbance of each sample / absorbance of control was calculated. Each experiment was performed > 5 times and obtained similar results. Statistical analysis was performed using Student’s t-test. Values are presented as means ± SEM versus control (*p < 0.05)

To determine the effects of LEF on human OSCC growth, cell proliferation, cytotoxicity, LDH, and migration assays were performed. Human OSCC cell lines SAS, Ca9-22, HSC2, and HSC3 were used. OSCC proliferation was assessed by the CCK-8 and colony formation assays. In the CCK-8 assay, LEF showed a less significant inhibitory effect at lower concentrations. However, it significantly inhibited the growth of all four cell lines at 100 µM (*p* < 0.05, Fig. 1a). In the colony formation assay, OSCC proliferation was inhibited by a relatively high dose (10 or 100 µM) of LEF (*p* < 0.05, Fig. 1b). On the wound-healing assay performed to evaluate the effect of LEF on cell migration, LEF dose-dependently inhibited cell migration in all OSCC lines (*p* < 0.05, Fig. 2a). LEF cytotoxicity was examined by measuring the LDH in the culture medium. As shown in Fig. 2b, the LDH amount in the culture medium was significantly reduced compared with that in the control group (*p* < 0.05); however, the amount did not correlate with LEF concentration. These results indicate that the cytotoxic effects of LEF concentrations < 100 µM are negligible, reflecting that LEF inhibits the proliferative and migratory ability of OSCC at noncytotoxic concentrations.

### LEF inhibits pyrimidine biosynthesis through DHODH suppression

**Fig. 3:**
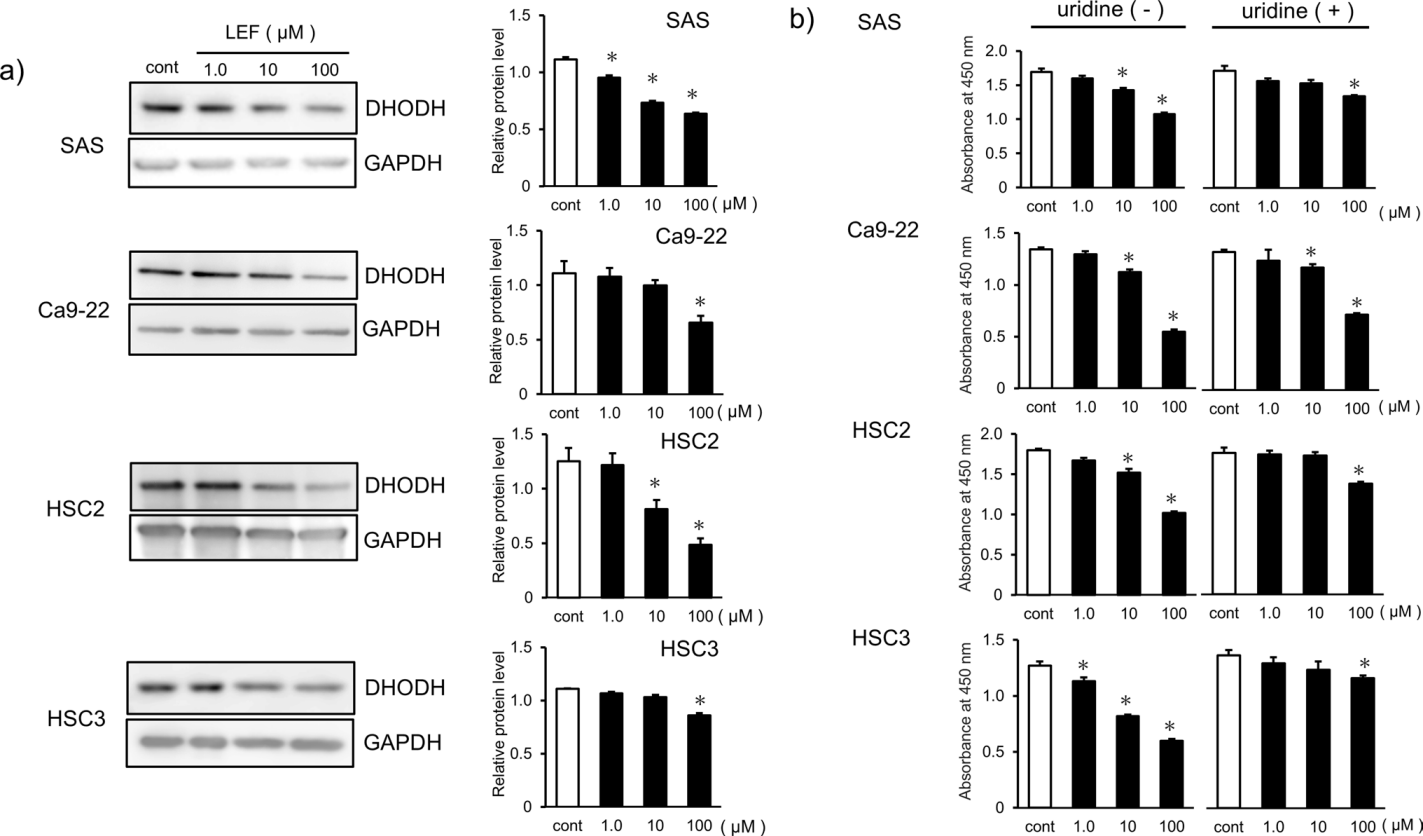
Suppression of DHODH expression by LEF in OSCC cells and restoration of cell growth by uridine supplementation. **a** Panels of Western blotting of DHODH and the internal control GAPDH in each OSCC cell line after treatment with the indicated concentration of LEF. The expression status of DHODH was evaluated quantitatively as a fold change compared with GAPDH. **b** Cell growth inhibition was restored by 100 µM uridine supplementation. Cell proliferation was assessed by the CCK-8 assay and quantified by measuring the absorbance at 450 nm wavelength. OSCC cells were incubated at the intended LEF concentration with or without 100 µM uridine for 72 h. Each experiment was performed > 5 times, and similar results were obtained. Statistical analysis was performed using Student’s t-test. Values are presented as means ± SEM (*p < 0.05)

LEF is a potent DHODH inhibitor and suppresses pyrimidine biosynthesis. Therefore, Western blot analysis of DHODH was performed to evaluate the pharmacological effect of LEF on OSCC lines. LEF significantly suppressed DHODH expression. In particular, SAS and HSC-2 showed decreased DHODH expression at relatively low LEF concentrations (< 10 μM) (*p* < 0.05, Fig. 3a). Then, to examine whether the restoration of pyrimidine biosynthesis rescues OSCC cell proliferation, uridine was introduced to the OSCC culture media, and the CCK-8 assay was performed. The results showed that uridine supplementation restored OSCC cell proliferation in 3/4 of the cell lines treated with 10 μM LEF (*p* < 0.05, Fig. 3b). These results indicate that LEF inhibits pyrimidine biosynthesis by DHODH suppression and exerts an inhibitory effect on OSCC.

### LEF suppressed in vitro angiogenesis in HUEhT-1 cells

**Fig. 4:**
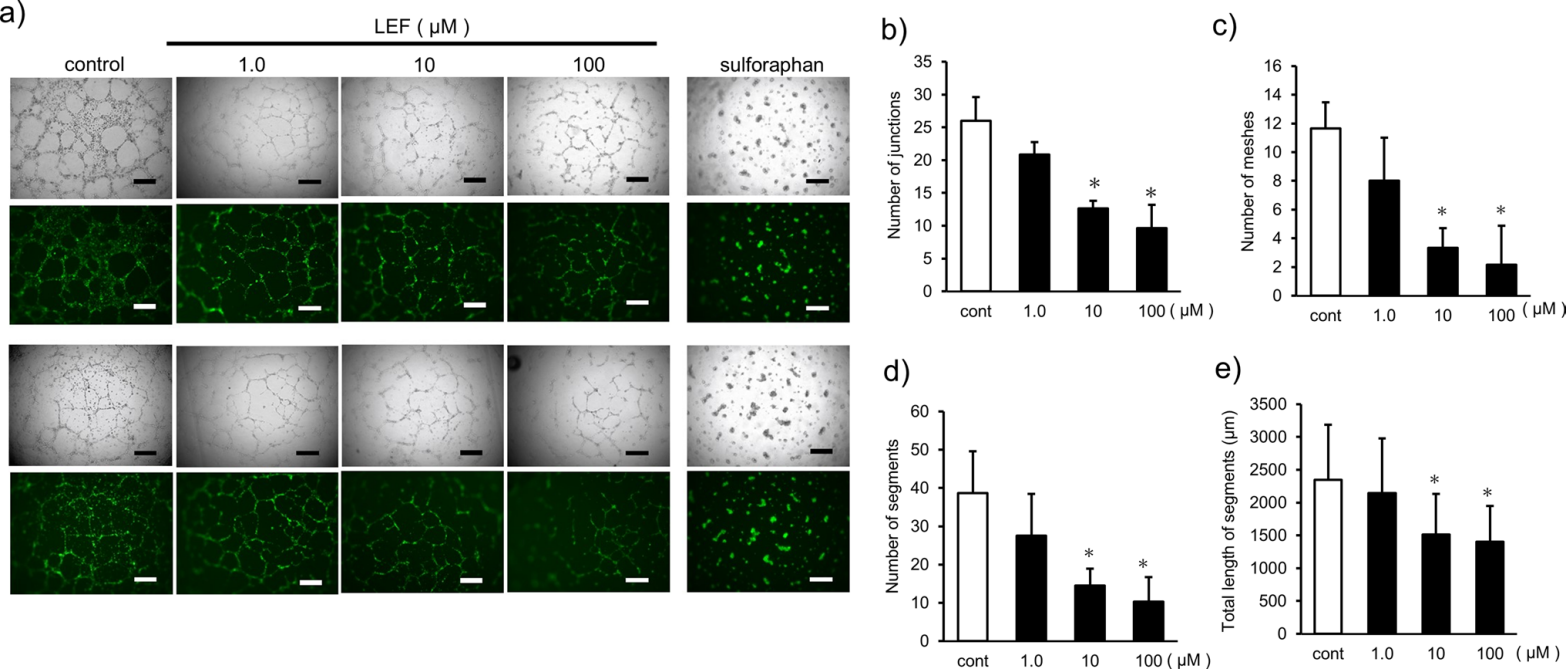
Evaluation of the inhibitory effect of LEF on angiogenesis in vitro*.* **a** Tube formation assay phase-contrast and fluorescence image panels. Two representative sites at different LEF concentrations are shown as phase-contrast (top) and fluorescence (bottom) images. Negative controls were cells treated with sulforaphane (10 μM). Bars in the panels indicate 500 μm. **b**–**e** Quantification of the number of junctions (**b**), number of meshes (**c**), number of segments (**d**), and total length of segments (**e**) by the Angiogenesis Analyzer for Image J. Each point was evaluated by measuring five different fields of view. Statistical analysis was performed using Student’s t-test. Values are expressed as means ± SEM of five fields (*p < 0.05)

As mentioned above, LEF inhibited tumor angiogenesis in some malignant tumors. In the present study, a tube formation assay was performed to assess the effect of LEF on angiogenesis in vitro. HUEhT-1 cells that were derived from human umbilical vein endothelial cells (HUVECs) and immortalized by the electroporation of hTERT were applied to this assay as endothelial cells. HUEhT-1 cells form vascular structures on extracellular matrix gels in the presence of angiogenic factors such as VEGFs. As shown in Fig. 4a, although the control group had a well-defined vascular structure, the LEF-treated group had disorganized and sparse vascular structure in a dose-dependent manner. LEF also dose-dependently decreased the objective outcomes of vascular structure, number of junctions, number of meshes, number of segments, and total length of the segments, and the measured parameters in the 10 and 100 µM administration groups significantly decreased compared with those in the control group (*p* < 0.05, Fig. 4b–e).

### Therapeutic effect of LEF on oral cancer in immunodeficient and syngeneic mice

**Fig. 5:**
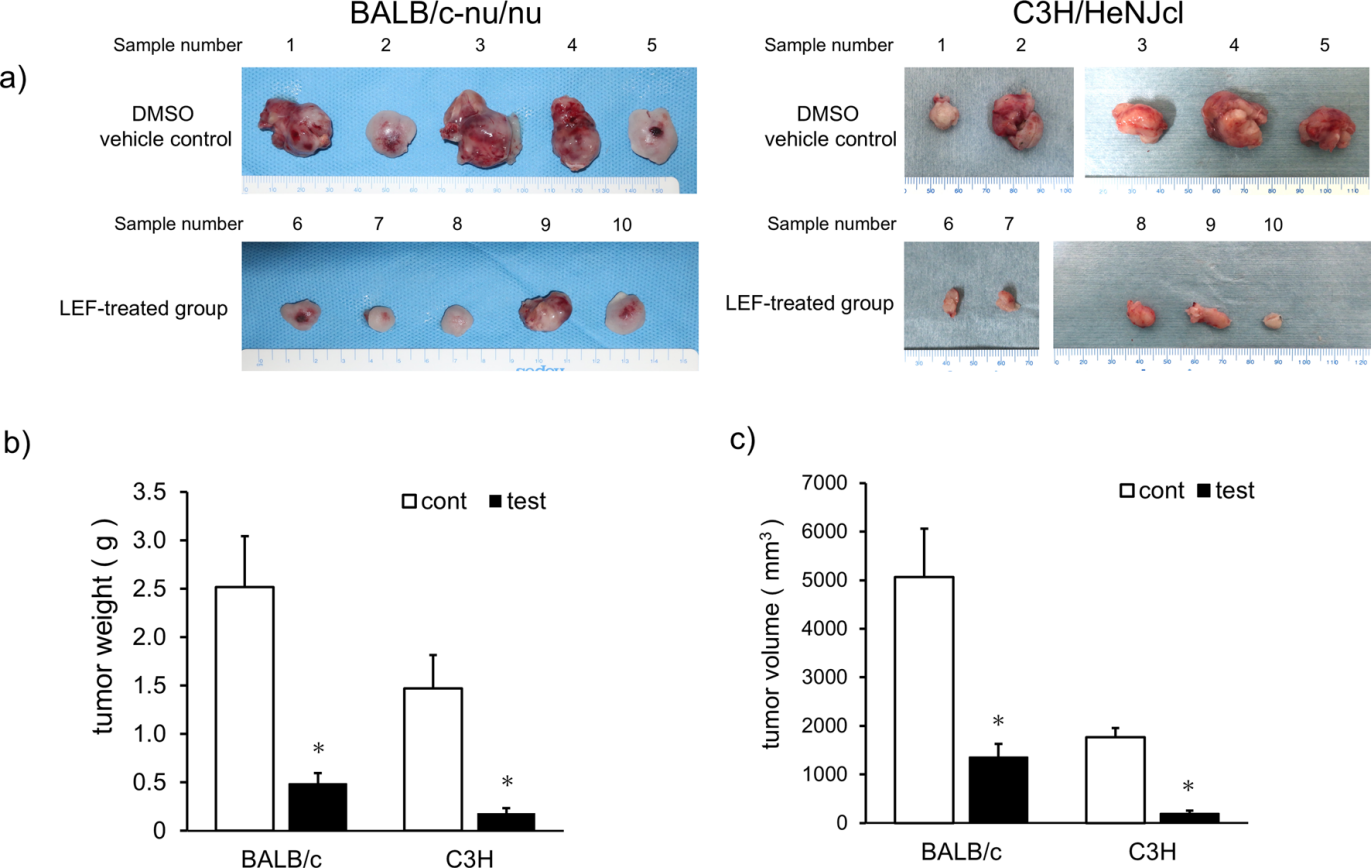
Anticancer effect of LEF on mouse OSCC allograft and syngeneic models. **a** Representative photos of the tumors of BALB/c-nu/nu (immunodeficient) and C3H/HeN Jcl (syngeneic) mice. Tumors of the control group (DMSO vehicle control; sample number 1–5) are shown in the upper panel, whereas that of the LEF-treated group (20 mg/kg/day for 14 days, LEF-treated group; sample number 6–10) are shown in the lower panel. **b**, **c** Weight (**b**) and volume (**c**) of the tumors extracted from BALB-c and C3H mice, respectively. White and black bars indicate the control and LEF-treated groups, respectively. Values are presented as means ± SEM (N = 5). Statistical analysis was performed using Student’s t-test. Significance was evaluated between the control and the test of each mouse (*p < 0.05)

Moreover, the anticancer effect of LEF on OSCC in vivo was investigated. Because LEF was a T-lymphocyte-specific immunosuppressant agent, we hypothesized that LEF might affect T-lymphocyte-dependent tumor immunity. Therefore, the in vivo antitumor effects of LEF on immunodeficient mice without T-lymphocytes (BALB/c-nu/nu mice) and syngeneic mice (C3H/HeN Jcl mice) with normal immune competence were investigated. The two types of mice were randomly divided into two groups of five each: a DMSO vehicle control group and a LEF-treated group. The LEF-treated group received an oral administration of LEF at a dose of 20 mg/kg/day for 14 days. This dosage was selected based on previous studies of LEF in renal cell cancer [24], non-small cell lung cancer [26], and bladder cancer [30]. Additionally, preliminary experiments confirmed that there was no weight loss observed in either the DMSO vehicle control group or the LEF-treated group of mice (data not shown). The cell line for transplantation was SCCVII, a mouse-derived OSCC. SCCVII cells were injected subcutaneously into the back of mice. After 2 weeks of inoculation, mice were sacrificed, and tumors were extracted. Consequently, the tumor was visibly smaller in the LEF-treated group than in the nontreated group (Fig. 5a). Syngeneic mice tumors were mostly smaller than those of immunodeficient mice. Furthermore, the immunodeficient and syngeneic mice in the LEF group had significantly reduced tumor weight (*p* < 0.05, Fig. 5b) and volume (*p* < 0.05, Fig. 5c) compared with those in the control group. These results indicate that LEF suppresses tumor growth in vivo without inhibiting T-lymphocyte-dependent tumor immunity.

### Inhibitory effect of LEF on DHODH expression and tumor vascularization in vivo

**Fig. 6:**
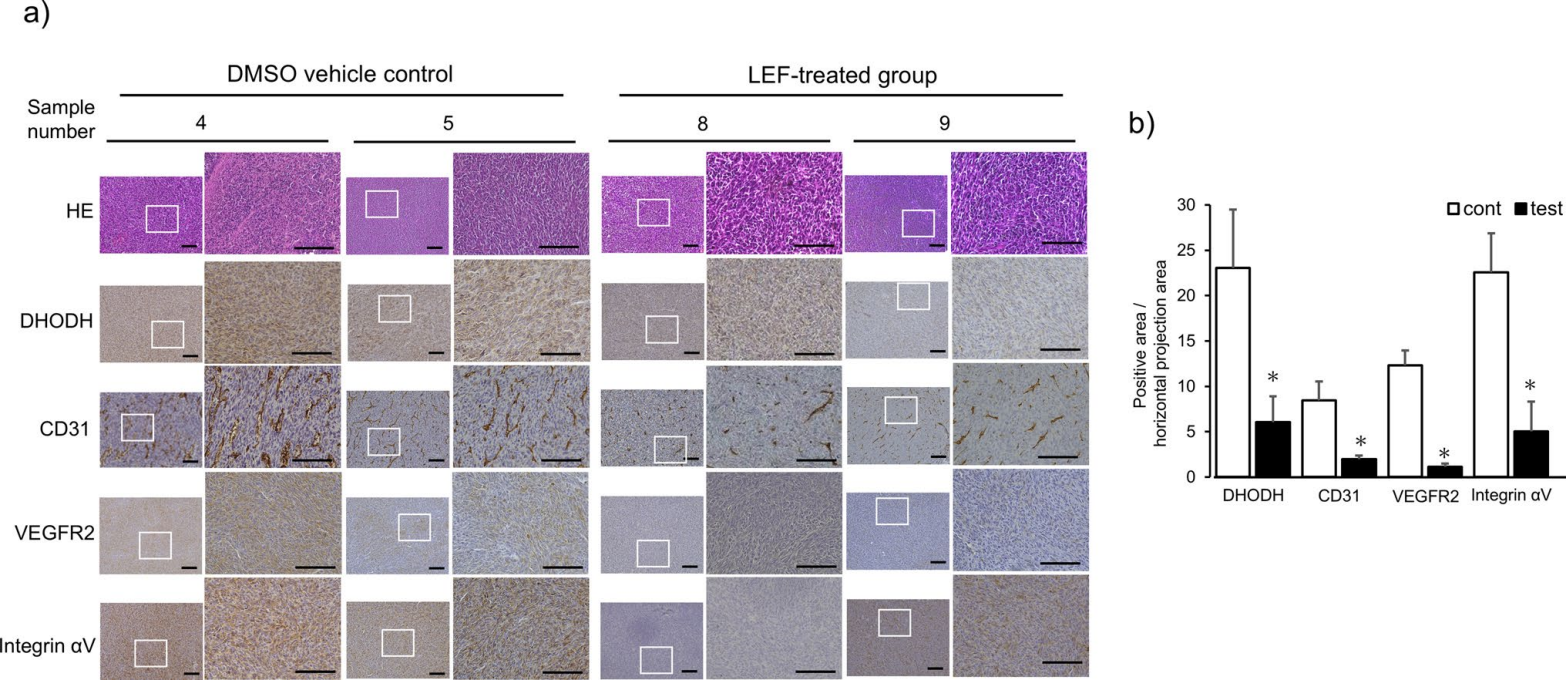
Immunohistochemistry study of tumor specimens of syngeneic mice. **a** Representative microscopic images of the specimen of syngeneic mice. HE staining and IHC staining for DHODH and the tumor vascular endothelial markers integrin αv, VEGFR2, and CD31 of the tumors from the control (DMSO vehicle control) and LEF-treated (LEF-treated group) groups. Ten fields of view were taken for each specimen, and images of representative fields of view (sample number 4, 5 as control and 8, 9 as test in Fig. 5) are shown on the left at low magnification and right at high magnification of the areas highlighted in white rectangle. Black bars in all panels indicated 500 µm. (**b**) Quantitative analysis of IHC positivity rates was conducted by calculating the ratio of the stained area of each marker, DHODH and vascular endothelial cells, to the horizontal projected area of the sections. This analysis was performed using BZ-X Analyzer software (BZ-H4A, Keyence Corporation, Osaka, Japan), with five sections randomly selected for each marker. Statistical analysis was performed using Student’s t-test. Values are expressed as means ± SEM of five sections (*p < 0.05)

To evaluate the effect of LEF on DHODH expression and tumor vascularization in vivo, IHC was performed on the extracted tumor samples from syngeneic mice. As shown in Fig. 6, DHODH expression was reduced in the LEF-treated group. The vascularity of tumors was also evaluated by staining endothelial cell markers CD31 and VEGFR2 and integrin αv, which were considered specific for tumor vessels. Each tumor endothelial cell marker was highly expressed in the control group, consistent with endothelial cells in the tumor tissue, whereas the expression of every marker was markedly decreased in the LEF-treated group. Quantitative analysis revealed that the positively stained area was significantly reduced with LEF administration (*p* < 0.05, Fig. 6b).

## Discussion

In this study, LEF demonstrated an inhibitory effect on OSCC growth and migration in vitro, and these effects were mediated by DHODH inhibition. Moreover, LEF inhibited tube formation of human endothelial cell lines in vitro, inhibited tumor growth in both immunodeficient and syngeneic mouse models, and markedly suppressed the angiogenesis in extracted tumors. In other words, LEF demonstrated an inhibitory effect on tumor progression by suppressing tumor angiogenesis without inhibiting T cell-dependent tumor immunity. Considering these results, LEF can be a candidate for drug repositioning as an anticancer agent targeting tumor angiogenesis against oral cancer.

Newer agents such as EGFR [9, 10] and immune checkpoint inhibitors [11–15] have shown efficacy in head and neck cancers, including oral cancer. Despite their emergence, conventional pyrimidine metabolism inhibitors such as 5-FU are still included in National Comprehensive Cancer Network guidelines as one of the first-line drugs in systemic therapy regimens [34]. 5-FU is an analog of uracil and inhibits DNA synthesis by direct incorporation into DNA. It also inhibits DNA synthesis through interference with thymidylate synthase activity and inhibition of thymine synthesis. Furthermore, 5-FU is metabolized to 5-fluorouridine triphosphate (FUTP), which is incorporated into RNAs instead of UTP, thereby interfering with mRNA translation [35]. Conversely, LEF is a DHODH inhibitor. DHODH is involved in the pyrimidine biosynthesis pathway, catalyzing the reaction of dihydroorotate to orotic acid. DHODH overexpression occurs in various malignancies, including hematopoietic and solid tumors [36, 37]. The activity of this biosynthetic pathway is increased in cancer cells with unlimited growth. In cases with rapidly increasing demand for pyrimidine biosynthesis, such as the proliferation of activated lymphocytes, the de novo pathway is more important than the salvage pathway. In response to the rapid demand for pyrimidine biosynthesis, cancer cell proliferation is also dependent on the de novo pathway [37]. In this study, DHODH expression was found in OSCC in vitro, which was dose-dependently suppressed by LEF, and the restoration of pyrimidine biosynthesis by uridine supplementation rescued the inhibitory effect of LEF (Fig. 3a, b). Moreover, immunostaining of tumor tissue samples from syngeneic mice with and without LEF treatment showed a clear correlation with DHODH expression. These results indicate that the growth inhibitory effect of LEF is more pronounced with decreased DHODH expression. Although its mechanism of action is different from that of conventional pyrimidine metabolism antagonists such as 5-FU, LEF is thought to exert its anticancer effects through the inhibition of pyrimidine biosynthesis.

As another anticancer effect of LEF, several basic research on other carcinomas revealed that LEF inhibits tumor angiogenesis. Chu et al. demonstrated that LEF inhibited tumor angiogenesis by inhibiting the sEphrin-A1/EphA2 system in both the bladder chemical carcinogenesis and bladder cancer xenograft models [31]. Mall et al. also demonstrated that LEF inhibited tumor angiogenesis in a colon cancer xenograft model [32]. Angiogenesis is one of the important mechanisms that contribute to oral cancer progression [38, 39]. Angiogenic factors, such as VEGF, PDGF, and bFGF, are overexpressed in various malignant tumors including oral cancer [40–42]. In this study, the effect of LEF on the vessel-forming capacity of endothelial cells was initially assessed by the tube formation assay on the extracellular matrix gel in vitro. As shown in Fig. 4, image analysis of the lumen-like network structures showed that LEF significantly dose-dependently inhibited vessel formation in endothelial cells. These results indicated the inhibitory effect of LEF on angiogenesis. However, tumor-specific vascular endothelial cells differ in properties and gene expression from normal vascular endothelial cells such as HUVECs. Tumor-specific molecules such as VEGFR2, integrin αvβ_3_, CD13 [43], and TEM8 [44] are specific to tumor vascular endothelial cells. Therefore, tumor-specific vascular endothelial markers were evaluated by IHC of specimens from syngeneic mice. The results revealed that the expression levels of VEGFR2 and integrin αvβ_3_, as well as the vascular endothelial marker CD31, were significantly suppressed in the LEF-treated group. These results indicate that LEF exerts an inhibitory effect on tumor angiogenesis induced by OSCC as in other malignancies. To our knowledge, there are no reports of LEF exhibiting an angiogenesis inhibitory effect in oral cancer. Nevertheless, EGFR, which is abundant in oral cancer [45], is known to be involved in both tumor angiogenesis and tumor growth [46]. Furthermore, it has been suggested that LEF can suppress the tyrosine kinase activity of EGFR [28]. This indicates that LEF may exert its angiogenesis inhibitory effect by inhibiting the tyrosine kinase activity of EGFR. Further research will aim to elucidate the mechanisms through which LEF exerts its angiogenesis inhibitory action in oral cancer. At present, no antiangiogenic agents have been established for OSCC; however, by clarifying the tumor vascular factors targeted by LEF, LEF may be used as a novel antiangiogenic agent.

As described above, LEF is a disease-modifying antirheumatic drug that inhibits T-lymphocyte proliferation and activity. Therefore, LEF might interfere with T-cell-dependent tumor immunity. In the present study, immunodeficient and syngeneic mice were used in the animal experiments. BALB/c-nu/nu mice are athymic and congenitally deficient in T cells. Therefore, analyzing the effect of T-cell-dependent tumor immunity is unnecessary. In contrast, the C3H mice used as a syngeneic model have normal immunocompetence. Therefore, the anticancer effects of LEF under T-cell-dependent tumor immunity conditions could be evaluated. As a result, the tumor size and volume were significantly reduced in the syngeneic mouse model than in the immunodeficient mouse model. Moreover, LEF concentration was significantly reduced in both immunodeficient and syngeneic models, and no difference in tumor reduction rate was found between the immunodeficient and syngeneic models. These results implied that T-cell-dependent tumor immunity may be responsible for tumor reduction. In addition, the anticancer effect of LEF should not be affected by T-cell-dependent tumor immunity. In head and neck cancer, the immune checkpoint inhibitors nivolumab and pembrolizumab have brought about a good prognosis in recurrent metastatic OSCC. Although the effect of LEF on tumor immunity remains to be elucidated, the combination of LEF with existing immune checkpoint inhibitors may generate new therapeutic strategies.

Regarding the antitumor effect of LEF on oral cancer, Ren et al. demonstrated that the inhibition of DHODH by LEF suppresses tumor growth through in vitro studies using human OSCC cell lines and in vivo studies using a human oral cancer xenograft mouse model [47]. The clear distinctions between the previous study by Ren et al. and our present study are as follows: First, we demonstrated the inhibitory effect of LEF on tumor angiogenesis in oral cancer. Second, we clarified how LEF, classified as a DMARD and broadly as an immunosuppressant, exhibited a high antitumor effect in a model with normal immune function. In light of these new findings, we recognize two areas that require further investigation: one is to elucidate the molecular mechanisms of LEF's effect on tumor angiogenesis, and the other is to clarify how LEF influences tumor immunity.

Limitations of this study include the difficulty in investigating the inhibitory effect of LEF on spontaneous OSCC. In addition, tumor vascular endothelial cell lines derived from OSCC tissues have not been established; thus, the effect of LEF on the tube-forming capacity of tumor vascular endothelial cells could not be investigated. Whether the in vivo experimental results obtained in this study can be achieved and maintained in humans at tolerated doses of LEF should be examined in a clinical trial.

LEF is already in clinical use, and data on its dosage, safety, and adverse events have been accumulated. Therefore, if LEF is effective against oral cancer, it may be introduced as a therapeutic agent as soon as possible by drug repositioning.

## Supplementary Information

Supplementary Material 1. Supplementary Fig. 1 showed the full-length Gel images of Fig. 3a

## Data Availability

All data supporting the findings of this study are available within the paper.
