# Supplementary material for: Anticancer effect of the antirheumatic drug leflunomide on oral squamous cell carcinoma by the inhibition of tumor angiogenesis
Source: Discov Oncol. 2025 Jan 16;16:53. doi: 10.1007/s12672-025-01763-5 (PMC11735718; doi:10.1007/s12672-025-01763-5)
Supplement: Supplementary file 1 — Supplementary Material 1. Supplementary Fig. 1 showed the full-length Gel images of Fig. 3a [file 12672_2025_1763_MOESM1_ESM.pptx]

## Slide 1
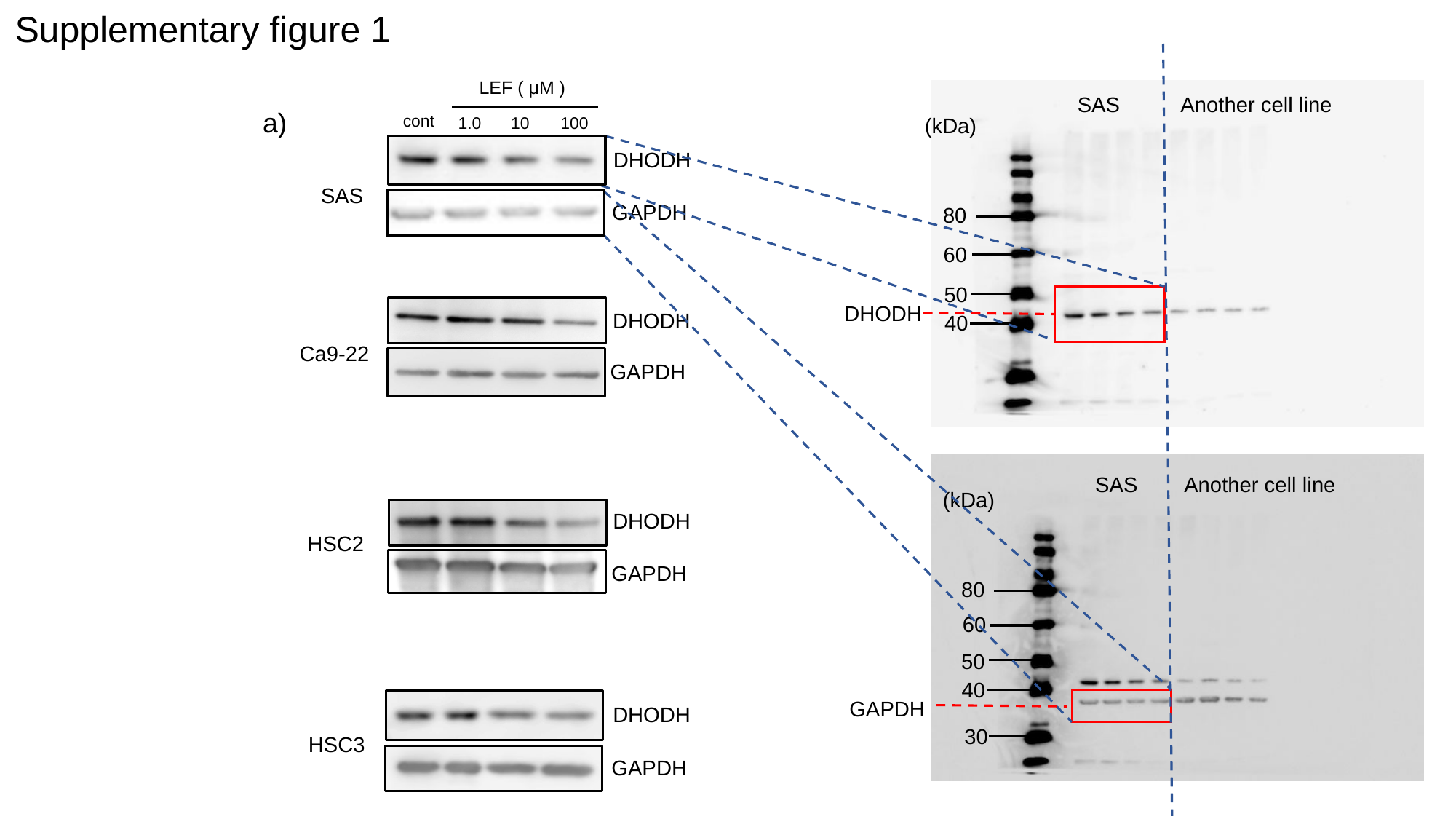

Supplementary figure 1
LEF ( μM )
Another cell line
SAS
# a)
cont
1.0
10
(kDa)
100
DHODH
SAS
GAPDH
80
60
50
DHODH
DHODH
40
Ca9-22
GAPDH
SAS
Another cell line
(kDa)
DHODH
HSC2
GAPDH
80
60
50
40
GAPDH
DHODH
30
HSC3
GAPDH

## Slide 2
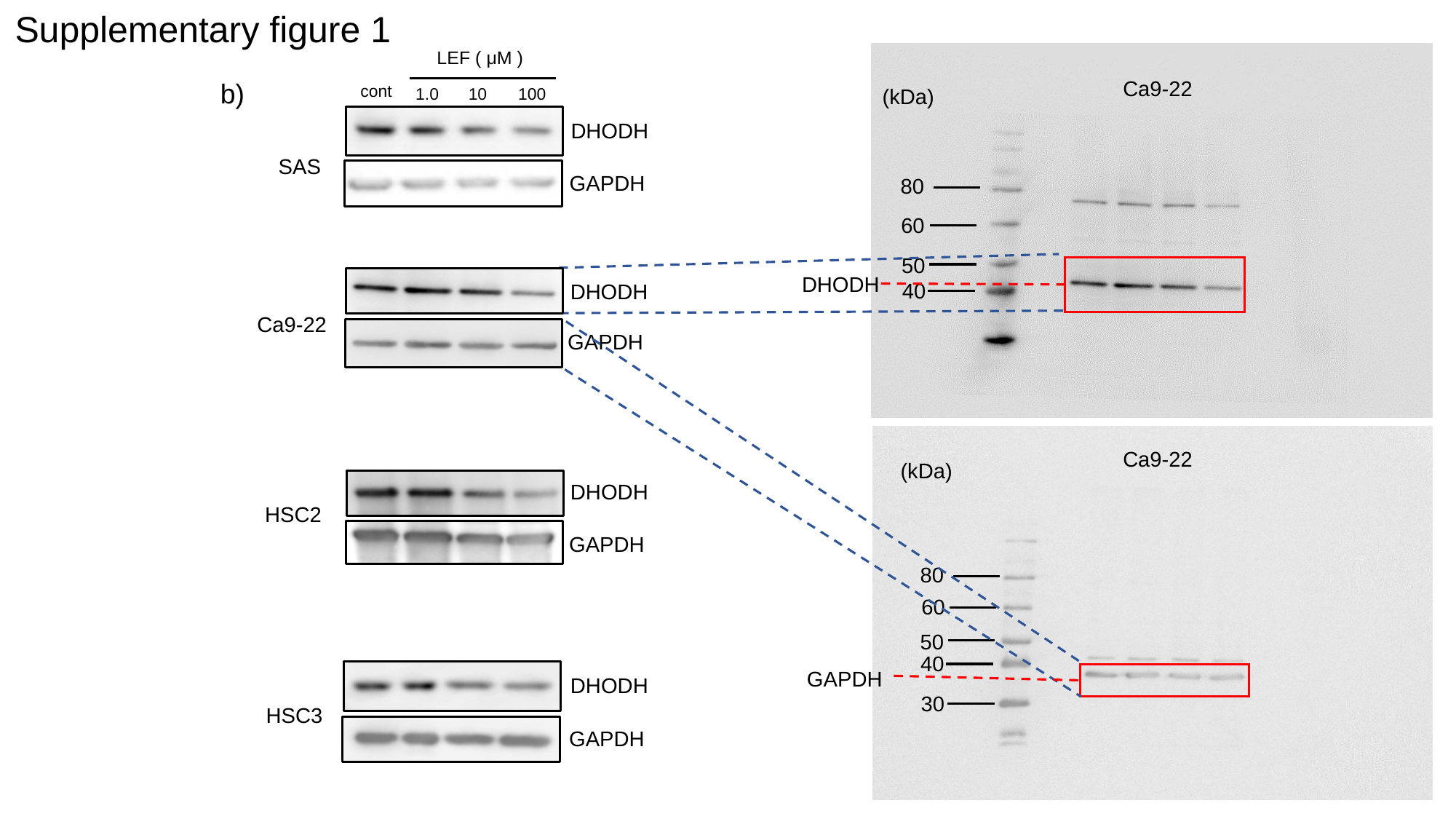

Supplementary figure 1
LEF ( μM )
Ca9-22
# b)
cont
1.0
10
(kDa)
100
DHODH
SAS
GAPDH
80
60
50
DHODH
40
DHODH
Ca9-22
GAPDH
Ca9-22
(kDa)
DHODH
HSC2
GAPDH
80
60
50
40
GAPDH
DHODH
30
HSC3
GAPDH

## Slide 3
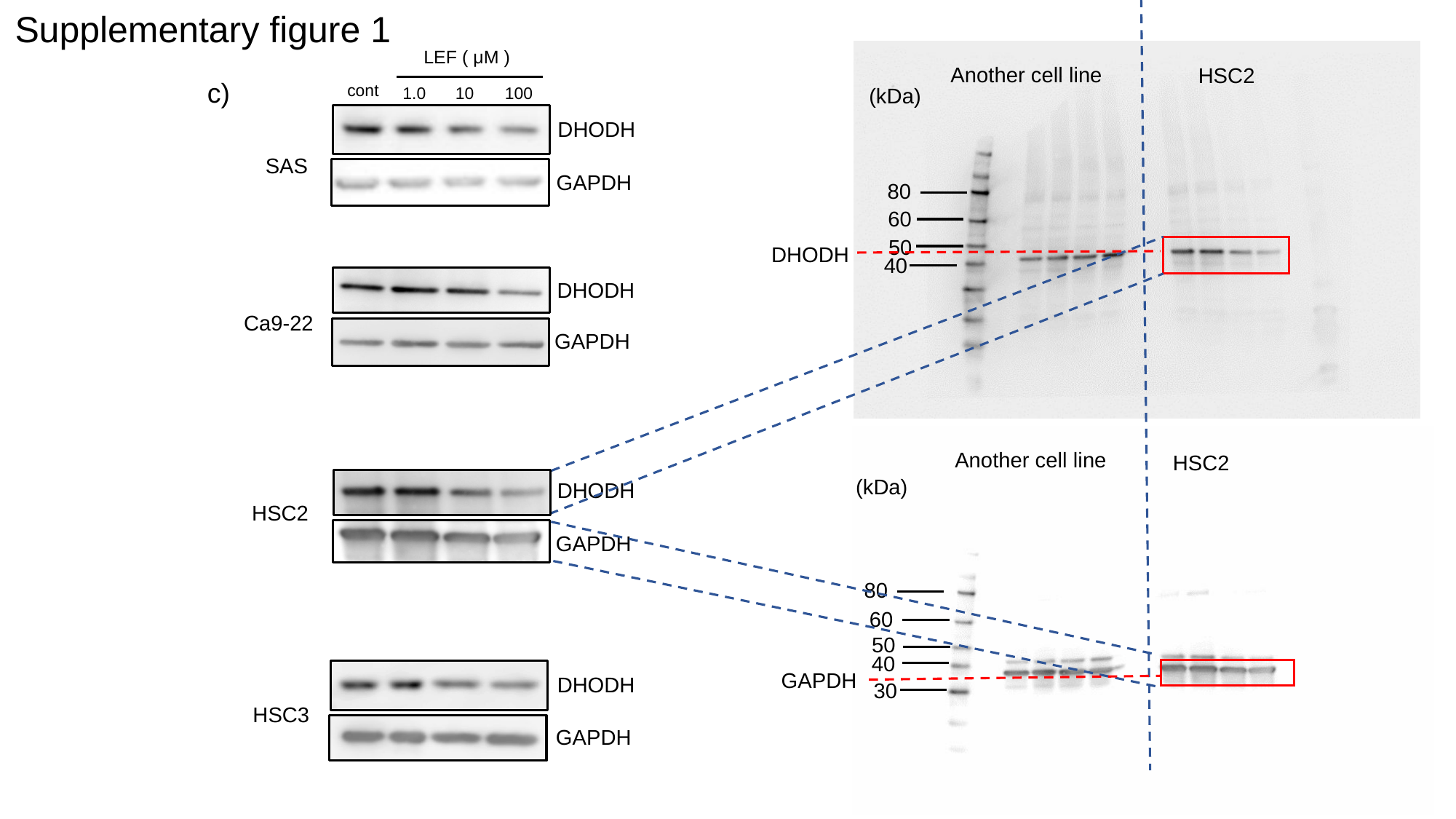

Supplementary figure 1
LEF ( μM )
Another cell line
HSC2
# c)
cont
1.0
10
(kDa)
100
DHODH
SAS
GAPDH
80
60
50
DHODH
40
DHODH
Ca9-22
GAPDH
Another cell line
HSC2
(kDa)
DHODH
HSC2
GAPDH
80
60
50
40
GAPDH
DHODH
30
HSC3
GAPDH

## Slide 4
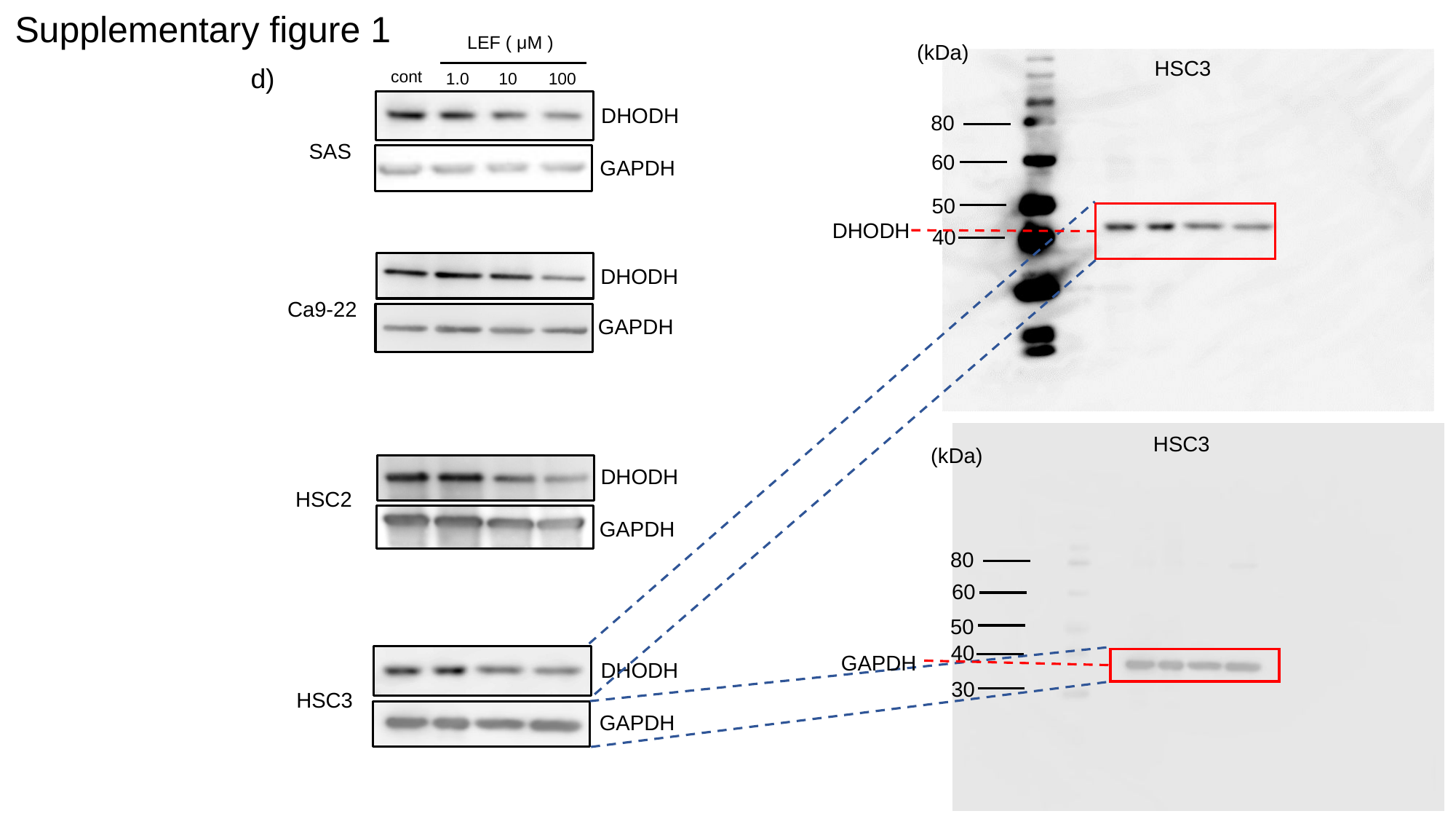

Supplementary figure 1
LEF ( μM )
(kDa)
HSC3
# d)
cont
1.0
10
100
DHODH
80
SAS
60
GAPDH
50
DHODH
40
DHODH
Ca9-22
GAPDH
HSC3
(kDa)
DHODH
HSC2
GAPDH
80
60
50
40
GAPDH
DHODH
30
HSC3
GAPDH
